# How white and black bodies are perceived depends on what emotion is expressed

**DOI:** 10.1038/srep41349

**Published:** 2017-01-27

**Authors:** Rebecca Watson, Beatrice de Gelder

**Affiliations:** 1 Department of Cognitive Neuroscience, Faculty of Psychology and Neuroscience, Maastricht University, The Netherlands; 2 Department of Psychiatry and Mental Health, University of Cape Town, South Africa

## Abstract

Body language is a powerful indicator of others’ emotions in social interactions, with positive signals triggering approach and negative ones retreat and defensiveness. Intergroup and interracial factors can influence these interactions, sometimes leading to aggressive or even violent behaviour. Despite its obvious social relevance however, the interaction between body expression and race remains unexplored, with explanations of the impact of race being almost exclusively based on the role of race in face recognition. In the current fMRI study we scanned white European participants while they viewed affective (angry and happy) body postures of both same race (white) and other race (black) individuals. To assess the difference between implicit and explicit recognition participants performed either an explicit emotion categorisation task, or an irrelevant shape judgement task. Brain activity was modulated by race in a number of brain regions across both tasks. Race-related activity appeared to be task- as well as emotion- specific. Overall, the other-race effects appeared to be driven by positive emotions, while same-race effects were observed for negative emotions. A race specific effect was also observed in right amygdala reflecting increased activation for explicit recognition of angry white body expressions. Overall, these results provide the first clear evidence that race influences affective body perception.

When we notice somebody approaching us from a distance we tend to prepare automatically for the interaction, whether this is reacting to an expression of joy or stepping back in defence to a display of anger. As highlighted in a number of well-documented cases, intergroup and interracial factors can complicate these interactions, sometimes leading to aggressive or even violent behaviour. Despite its obvious social relevance however, the impact of race on social interaction and emotion processing has so far received scant attention within the field of cognitive neuroscience. Furthermore, the few explanations of the impact of race on social interactions are almost exclusively based on studies of face perception.

Recently there has been growing interest in the neural mechanisms engaged when people process race, and how this may influence perceptions and behaviours. Studies using faces have shown that we are generally adept at recognising emotions across culture, but tend to do so more accurately in members of the same cultural group, paralleling the now well-documented ‘other race effect’ found for face identity recognition^1^. Furthermore, biases in emotion perception have often been reported – for example, the perception of other race faces as more hostile or aggressive (typically demonstrated for black vs. white face stimuli, as seen by white participants)^2^, a perception which is correlated with behavioural measures of implicit bias^3^.

An increasingly important literature addresses the issue of group belonging^4^,^5^,^6^Although *de facto* there is an overlap between issue of ungroup-outgroup behavior and race issues, these may ultimately be different theoretical questions. Our study focuses on race perception in a group of participants that has no experience with the “other” race. As a consequence there was no group dynamics that may have led to the psychological construction of the other race as the outgroup. In the absence of such pre-existing dynamics, the attitude to the own race group may be either positive or negative, but attitude to the other race has neither a positive nor a negative valence.

Granted that studies are limited and have generally considered race effects using faces, there is now evidence of a network of brain regions sensitive to race, with each of these regions thought to be involved in different aspects of race processing and evaluation. For example, two brain regions associated with face perception – the lateral fusiform gyrus, specifically the ‘fusiform face area’ (FFA)^7^, and the posterior cingulate cortex – tend to show a greater response to own race vs. other race individuals^8^,^9^,^10^. This enhanced activity is suggested to reflect greater experience with racial in-group members, a familiarity effect linked to processing expertise. In contrast, heightened responses to other-race individuals have been consistently linked to the amygdala^11^,^12^. This subcortical structure is well evidenced as playing a role in emotion processing^12^,^13^,^14^, and enhanced activation may be linked more with implicit evaluation processes, particularly the arousal of negative affect^10^,^11^,^15^,^16^,^17^,^18^. Besides the amygdala, the anterior cingulate cortex, and dorsolateral and ventrolateral prefrontal cortex also show a heightened response to other-race faces^10^,^11^,^19^,^20^. However, this is proposed to be more a result of emotion conflict and regulation processes: that is, the conflict between conscious and subconscious attitudes, and the resulting engagement of regulatory mechanisms in order to control implicit, subconscious, and often negative racial associations.

The impact of race on daily social interaction is not only limited to people who are close by us. We are well able to recognise affective information from bodily gestures and postures seen from a distance, and we react in an adaptive fashion even when going about our daily business often without waiting to see more specific personal details^21^,^22^. Indeed, the notion that the whole body displays the adaptive function of emotions was already at the core of Darwin’s views^23^. Over the past decade there has been increasing interest in the body as a pre-eminent social signal: it is now well established that we are easily able to recognise affective information from bodily gestures and postures and there is strong evidence for a specific neurobiological network of emotional bodily expressions^22^,^24^,^25^,^26^. Whole body expressions can be recognized from far away even when we cannot yet see the expression of the face or recognize the person^22^, and this makes bodies intriguing stimuli for better understanding the impact of race on emotion and social interaction. We know from previous studies that perceiving whole body expressions of emotion involves activity in brain areas that partially overlap with the action observation network. Major structures include premotor cortex, left inferior parietal lobule (IPL), amygdalae, bilateral inferior frontal gyrus, bilateral insula, right superior frontal gyrus and left middle occipital gyrus, along with structures such as the cingulate gyrus and cerebellum and primary motor cortex (precentral gyrus), all to some extent a function of the specific taks and stimuli.

The aim of this study was to investigate the effect of race on the neural network of emotional body perception. We used functional magnetic resonance imaging (fMRI) to measure participants’ cerebral oxygenation level while they viewed own-race and other-race affective body stimuli. As race differences may exert an implicit influence that may not be manifest in explicit recognition of the emotion, participants performed either an explicit emotion categorisation task, or an irrelevant shape detection task. We predicted that there would be reliable neural differences associated with other-race processing, that this may be dependent on whether this processing was explicit or implicit, and furthermore, whether the expression was of a positive or negative emotion.

## Results

### Whole brain analysis

Averaged across task, we observed a significantly overall higher response to black bodies as compared to white bodies in one cluster in the left inferior parietal lobule (IPL). No regions responded more to white bodies compared to black bodies, averaged across emotion. However, analysis of emotion-specific effects indicated that the right fusiform gyrus showed more activation to white angry bodies than black angry bodies, whereas the reverse comparison elicited no significant activation. In contrast, a large network of parietal, frontal and occipital and occipito-temporal regions, along with the left insular cortex, showed a heightened response to black happy bodies as compared to white, but we observed no regions that were significantly more activated by white happy bodies.

Analysis of task-specific activation showed that in the emotion categorisation task, black vs. white bodies (averaged across emotion) activated the right insula, and left superior and medial frontal gyri; furthermore, in this task, white bodies elicited more activity in the right parahippocampal gyrus, extending to the amygdala and hippocampus, as compared to black bodies. No regions responded more to black angry bodies as compared to white angry bodies, and vice versa. However, the bilateral inferior frontal gyrus, bilateral insula, right superior frontal gyrus and left middle occipital gyrus activated to black happy bodies vs. white happy bodies, whereas the reverse contrast elicited no significant activation.

**Figure 1:**
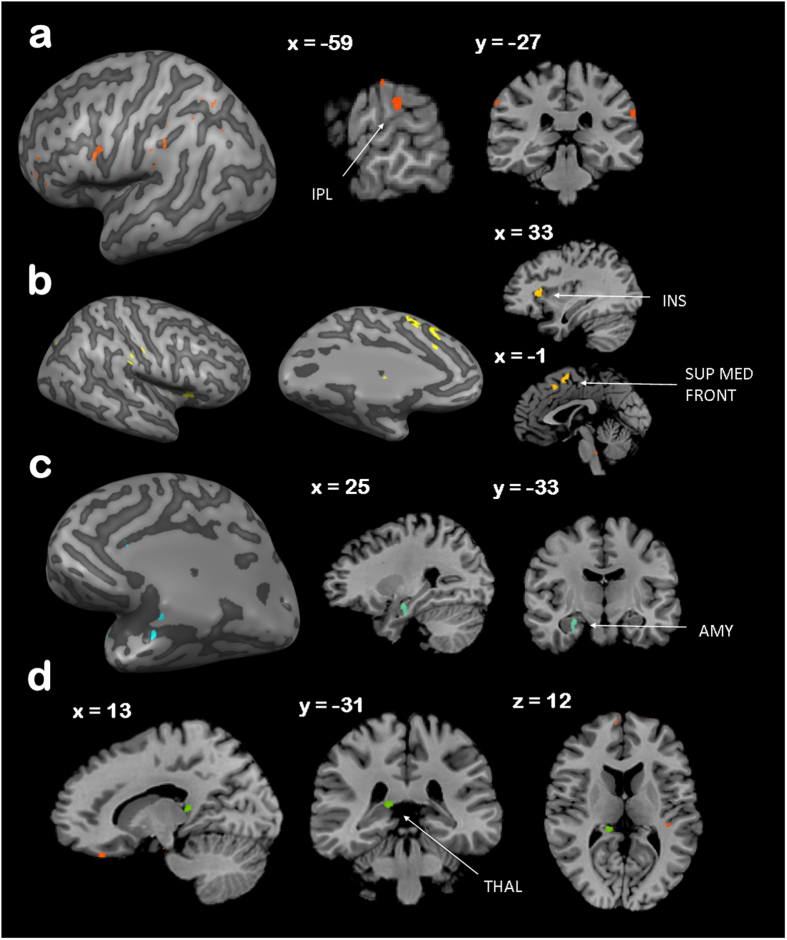
Race effects within a whole brain analysis; (**a**) Black vs. White body stimuli, averaged across task and emotion; (**b**) Black vs. White body stimuli, within the emotion categorisation task and averaged across emotion; (**c**). White vs. Black body stimuli, within the emotion categorisation task and averaged across emotion; (**d**). Black vs. White body stimuli, within the shape categorisation task and averaged across emotion.

Within the shape judgement task, only the right thalamus was significantly more activated by black bodies. No regions responded more to white vs. black bodies. Furthermore, the right precentral gyrus and fusiform gyrus, and left posterior cingulate, precuneus, medial frontal gyrus, middle frontal gyrus, middle occipital gyrus and superior temporal gyrus all showed activity for the contrast white angry bodies vs. black angry bodies, however the reverse contrast did not highlight any clusters. Finally, a large network of parietal, temporal and occipital regions, along with structures such as the cingulate gyrus and cerebellum showed more activity to black happy bodies vs. white; the converse contrast showed no significantly activated clusters. All whole-brain analysis contrasts can be seen in Tables 1, 2 and 3; contrasts are also illustrated in Fig. 1.

### Region of interest analysis

**Figure 2:**
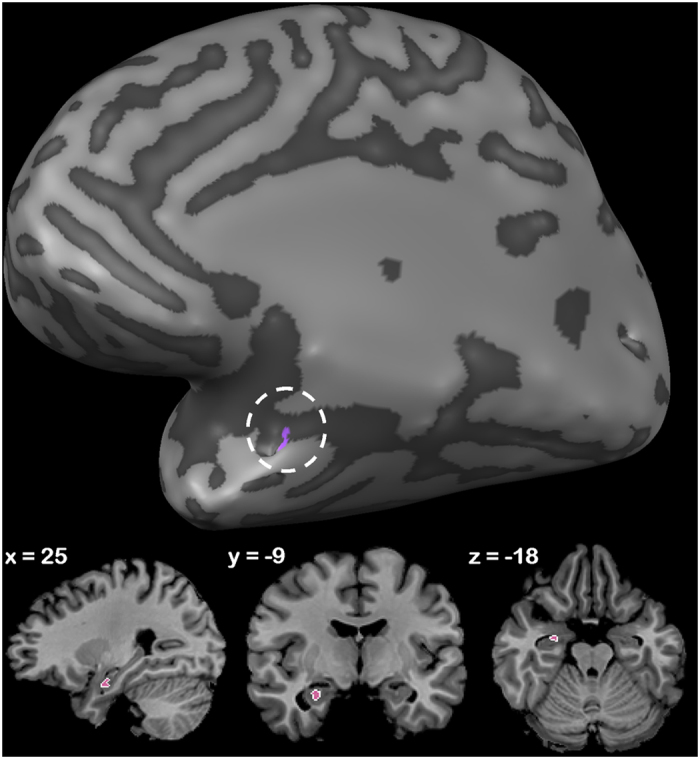
Race effects within a targeted region of interest within the right amygdala. White vs. Black body stimuli averaged across task and emotion.

A region of interest analysis limited to the bilateral amygdalae showed more activity in the right amygdala to white vs. black bodies, both averaged across task and within the emotion categorisation task. Activity in the amygdala is detailed in Table 4 and Fig. 2. No correlation was found between participants’ self-reported empathy/anxiety, as measured by the Interpersonal Reactivity Index (IRI) and State-Trait Anxiety Inventory (STAI) respectively (see Methods), and race related amygdala activity.

### On-line behavioural data

Mean condition accuracies (±standard deviation (S.D.)) for the black angry body, black happy body, white angry body and white happy body conditions within the explicit emotion categorisation task were 72.53 (±4.45) %, 93.41 (±2.50) %, 92.31 (±2.68) % and 94.51 (±2.29) % respectively. The mean condition accuracy (±S.D.) for the shape categorisation task was 97.99 (±3.85) %.

## Discussion

Here we confirmed that race does indeed influence brain responses to images of affective body expressions. We found that brain activity was modulated by race in a number of brain regions across both tasks, and that race-related activity appeared to be task- as well as emotion- specific. Overall, the other-race effects appeared to be driven by positive emotions, while same-race effects were observed for negative emotions. We discuss other-race, then same-race effects in turn below.

Firstly we found a network of regions sensitive to other-race, black bodies. Within a whole-brain analysis, one cluster within the left inferior parietal lobule (IPL) responded overall more to black affective, as compared to white affective bodies when averaged across task; additionally, in *explicit* emotion categorisation conditions, the contrast of black vs. white bodies elicited activation in clusters spanning the insula, superior frontal gyrus and medial frontal gyrus, whereas in the *implicit* task this contrast evoked activity only in the thalamus.

The IPL, insula, superior and medial frontal gyri are all regions which have been implicated in both affect processing^27^,^28^,^29^,^30^,^31^ and empathy – the ability to understand and share others’ emotions, feelings and perspective^32^,^33^,^34^,^35^. Furthermore, both the IPL and insula also appear to be modulated by both race and group membership. For example, two recent studies have described heightened IPL activation when participants imitated and observed neutral gestures of own-race versus other-race actors^36^,^37^. This result relates also to a previous investigation, which explored group membership effects on brain activity^38^. This study found that participants showing a behavioural in-group bias exhibited greater activity in the IPL when observing in-group members performing an action, as compared with members from the out-group. Regarding insular activity, it has been found that helping an in-group member perceived to be in pain was predicted by anterior insula activation^39^; similarly, viewing painful stimulations applied to racial in-group faces elicited increased activation in the insular and inferior frontal cortex (along with the ACC) in both Caucasian and Chinese participants^40^. Interestingly, these results are in contrast to ours, where we found a specific other-race, or out-group effect in these regions. However, there are a number of important differences in our study: firstly, we used whole body expressions as opposed to neutral hand actions or faces; secondly, these whole body expressions were expressive. Finally, we did not manipulate in-group vs. out-group membership. Our participants belonged to a homogeneous population with no real life experience with black populations. Therefor we have no reason to assume that they perceive the black body expressions as representing the out-group. More specifically, IPL is a crucial hub in a number of action and emotion related processes associated with body emotion expressions. The IPL in particular has been linked to affective body processing^41^,^42^ and, as part of the dorsal processing stream, plays an important role in action-observation-execution^43^. Therefore, the heightened activity to these other-race bodies may be more related to an increase in action-related perception. The right IPL responds to salient environmental events, such as target detection^44^,^45^. It has also been shown that right IPL is involved in maintaining attention^46^. These two functions may make the IPL particularly suitable for a role in phasic alerting, when behavioural goals need to be adapted in response to a salient stimulus. This line of argument is consistent with evidence from monkey research showing connections of the subcortical structures in superior colliculus, hippocampus and the cerebellum to specific subregions of the IPL^47^. Another aspect of the results that stresses the role of ethnicity is related to the positive activations obtained here for black happy bodies in areas related in the literature to empathy. The cingulate gyrus figures prominently in fMRI studies of empathy and social cognition^48^,^49^ but also in research on reward in relation to social factors^50^.

In view of the role of insula and its possible role in emotional awareness, it is worth nothing that the insula activation is obtained in revealing what is specific for explicit emotion recognition, but insula does not show in the implicit task, consistent with Also worth noting is the strong bilateral activation in precuneus. This brain area has been associated with social perception. More speculatively, this activation may be linked to a recent study identifying precuneus as closely related to happiness^51^. This makes for an interesting result as this interpretation suggests that empathy with the positive reward experienced by others comes easier and more spontaneously when one has a neutral attitude and no history of personal involvement with them. It is worth nothing again that these activations are prominent in the shape recognition task condition and are thus much less likely to express explicit cognitive processes.

Secondly, we looked at same-race effects. In a whole brain analysis we saw that averaged across task, no regions showed heightened activation to white bodies; however, when task-specific effects were taken into account we saw that in the explicit emotion categorisation task, a cluster spanning the amygdala, hippocampus and parahippocampal gyrus was more active. A region-of-interest analysis within the amygdalae indeed showed a heightened response to same-race bodies averaged across tasks, and within the emotion categorisation task, which was restricted to the right amygdala. The amygdala has been previously implicated in the perception of affective body expressions together with fusiform gyrus^52^,^53^. Furthermore, activity in this region tends to be more pronounced during conditions of explicit emotion categorisation. For example, a study by Habel *et al*.^54^ found that when participants performed an explicit emotion recognition task compared to an implicit (age discrimination) task on emotional faces, stronger activation occurred during explicit emotion recognition^54^. Derntl *et al*.^55^ also note that although stronger reactions to out-group stimuli (mainly faces) has been observed, factors such as task instruction may modulate this activity^55^.

Regarding the role of the amygdala in race perception, the vast majority of studies using faces as stimuli found greater amygdala BOLD activity to other-race as compared to own-race individuals^10^,^11^ which is in contrast to our results. At this point, it should be highlighted that although a stronger response to other race individuals is generally observed, there do exist nuances and inconsistencies within the literature. For example, one study found that there was no overall effect for black vs. white faces when eye gaze was indirect^56^ and another found that black American participants actually showed greater amygdala activity to own-race faces^57^. Furthermore, Chiao *et al*.^58^ observed that Japanese and Caucasian individuals showed greater amygdala activation in response to own-race faces expressing fear^58^. There also appears to be variability in amygdala reactivity across both individuals and groups which may be related to differences in implicit racial attitudes. For example, Phelps *et al*.^15^ did not find an overall BOLD signal difference between black and white faces in white American participants, but instead reported that the difference correlated with implicit measures of pro-white race preference across participants^15^. It may be that the use of groups of different nationalities and cultures strongly influence the neural response to ‘out-group’ or ‘other-race’ individuals. Specifically, it could be that certain races are not as salient in one cultural group, as compared to another. Notably, Van Bavel *et al*.^59^ write that when race is the most salient social category, the amygdala may respond to members of those groups associated with novelty or threat^59^. However, if this is not the case, the amygdala may be responsive to members of groups that are the most relevant, which can also include the in-group. This could indeed be the case in our tested group.

Significantly, both the observed significant same-race and other-race activations appeared to be emotion specific. We found that a number of regions responded specifically to negative (i.e., angry) bodies of the same race, compared to those of the other, whereas no regions emerged for this contrast when we considered positive emotions. Averaged across task, we found that the fusiform gyrus showed heightened activation for white angry bodies. Across both tasks there is an emotion specific effect in right fusiform gyrus triggered by angry white bodies. Increased fusiform gyrus activity may reflect higher familiarity with white than with black bodies without actually being evidence for race specific processing, as seen for faces^60^. But the fact that in the emotion specific contrast this activation is specific for angry bodies points in the direction of emotion based processing involving connections between fusiform gyrus and amygdala which then fits well with the increased amygdala activation for black angry bodies.;

Although no effects emerged in the explicit categorisation task, within the implicit processing task angry bodies evoked activity in the precentral gyrus, precuneus, posterior cingulate, medial frontal and middle frontal gyrus, along with the middle occipital gyrus. However, for black body expressions it was the positive, happy expressions, which appeared to drive the observed effects. Concerning the pattern of activations triggered by white angry bodies, the precentral gyrus and medial prefrontal activations are particularly important. The latter is well known for being crucially involved in fear reactions^61^ as presumably triggered here by the white bodies. In the same vein, we have systematically observed activations of precentral gyrus for threatening body expressions, most recently^21^,^22^. Thus medial prefrontal and precentral gyrus are two major areas associated with fear behaviour and our findings are consistent with a large literature. What is novel though is that they are here specifically associated with white anger body expressions.

This overall pattern of heightened activation for angry white and happy black bodies is perhaps in contrast to what one might expect, in that some previous studies have highlighted that other-race individuals can often be perceived as threatening, and therefore we might have predicted that the angry black expressions may have been more salient than angry white expressions. However, behavioural responses indicated that this was not the case: as mentioned previously this may be dependent on the particular cultural group tested, and whether there has been real life exposure to other-race (in this case, black) individuals and whether this has been of a negative nature. Taken together our results reveal that both same and other-race bodies seem to trigger action perception related activity, but in different brain regions as a function of the emotion expressed. Specifically, it seems that white (same-race) angry bodies elicited activity linked to an adaptive ‘retreat’ action, whereas black (other-race) happy bodies were possibly more linked to approach.

Some minor questions concern the possible impact of differences in ratings of the stimuli and the absence of correlations with questionnaire results. About the latter, there may be different explanations for the absence of correlation between the trait anxiety and amygdala activation. One is that the measure of trait anxiety in this questionnaire does not relate to that task and the contrast we considered here. Indeed, such direct relations are a matter of debate with very little evidence so far for either a positive relation with state anxiety^62^ and a negative with trait anxiety^63^. Another possibility is that our sample although substantial for an fMRI study, is not large enough to obtain stable correlations (the study by Bishop *et al*. used N = 50). We expected that the task variable that was used and the use of both an implicit and an explicit task would catch more theoretically relevant dimensions of the activations.

However, another question is related to the behavioral ratings and their possible impact on the observed pattern of activations. In our sample the recognition of emotions is easier for white compared to black bodies. But the pattern of activations reported here is not likely to reflect simply this difference in recognition rates. We note that behavioral performance in the explicit task is near ceiling for white and black angry bodies but that the pattern of brain activations is radically different. Furthermore, the clearest results with respect to race are obtained in the implicit condition where no recognition of the emotion was required and accuracy in the shape task was at ceiling for all stimulus categories.

The finding that emotional reactions are not simply a function of skin colour, but also that there seems to be a balance between skin colour and emotion expressed raises the issue of the biological, social and cultural basis of body perception and its influence on how people interact. At this point we note that the participants in this study come from a relatively homogeneous social-cultural population with virtually no real life experience to black populations. Previous studies have mostly used faces and racially more complex populations like the USA. Further research needs to clarify whether the current pattern of results generalizes to different sociocultural groups.

## Methods

### Participants

Data from 21 white European participants (8 male, mean age ± S.D. = 22 ± 3.22 years) was analysed for this study. Participants were all studying in Maastricht, The Netherlands at the time of testing and were currently resident in The Netherlands, Germany or Belgium. The participants represented a homogeneous ethnic group all recruited from the local student population. None did have any personal experience with other ethical groups, they had not previously performed experimental tasks where recognition of body expressions was requested and they had no experience or professional practice involving whole body movements other than occasional sports activity and casual recreational dancing.

Each was paid 15 Euros for completing the study and written informed consent was provided prior to the start of the protocol. The session took place at the neuroimaging facility at Maastricht University, and the study was approved by the Ethics Committee of Maastricht University, conducted in accordance with the approved guidelines.

### Stimuli

Stimuli were images of angry and happy body postures, of both black African ‘other-race’ and white European ‘in-race’ ethnicity. Ten upright, white affective body stimuli (i.e., five males each expressing anger and happiness) were selected from a set previously validated by our laboratory^64^. A set of black body expressions was obtained by following the same procedure used for the white set. Black African participants, all resident in Cape Town, South Africa, were instructed by a local collaborator to imagine a range of daily events and show how they would react to them (e.g., anger, happiness, disgust etc.) with their whole body. Photographs were captured with a Nikon V1 35 mm camera using a Nikon 30–110 mm lens on a tripod, under studio lighting, ensuring that the pictures showed the entire body, including hands and feet. Ten white European participants were then asked to categorise the emotion of a shown photograph, with options including neutrality, anger, fear, sadness, disgust and happiness. Based on these results, we chose five male identities, with each identity expressing both anger and happiness for inclusion in the final experiment. All emotions were recognised with 70% accuracy and upwards. Pictures were edited using Adobe Photoshop CC 14 software (Adobe Systems Incorporated) in order to mask the faces using an averaged skin colour; thus, no affective information was presented in the face. This resulted in a stimulus set of 20 affective bodies (2 race (Black, White) x 2 emotions (Angry, Happy) x 5 identities). Finally, we assessed the valence and intensity of our stimuli in two offline experiments. In Experiment 1, 15 participants saw each of the 20 stimuli three times in a randomised order. Each presentation lasted one second, after which they rated the valence of the expression on a 7 point scale (1 = very negative, 7 = very positive) and the intensity (1 = not at all intense, 7 = very intense). Average valence ratings for each category (mean ± S.D.) were as follows: black angry bodies = 3.19 ± 0.76; black happy bodies = 4.94 ± 0.81; white angry bodies = 2.19 ± 0.61; white happy bodies = 5.71 ± 0.55). Average intensity ratings for each category (mean ± S.D.) were as such: black angry bodies = 3.83 ± 0.86; black happy bodies = 4.01 ± 1.24; white angry bodies = 4.58 ± 1.21; white happy bodies = 4.89 ± 1.14). Experiment 2 was identical to Experiment 1, with the only difference being that bodies were presented for only 50 ms. Average valence ratings for each category (mean ± S.D.) were as follows: black angry bodies = 3.69 ± 0.55; black happy bodies = 4.97 ± 0.69; white angry bodies = 3.14 ± 0.80; white happy bodies = 5.64 ± 0.43). Average intensity ratings for each category (mean ± S.D.) were as such: black angry bodies = 3.72 ± 0.81; black happy bodies = 4.21 ± 1.18; white angry bodies = 3.89 ± 0.86; white happy bodies = 4.91 ± 1.09).

#### Complementary questionnaires

Participants received complementary questionnaires. The IRI^65^ measures four different aspects of dispositional empathy: perspective taking, empathic concern (feelings of sympathy and compassion for other people upon seeing discomfort in others), personal distress (feelings of distress in the individual upon seeing discomfort in others) and fantasy (the possibility to transfer oneself to a fictional situation). Participant rate 28 statements on a 5-point scale (1-Does not describe me well/5 - Describes me very well). The STAI measures trait and state anxiety levels in the individual^66^. Participants rate 20 items for each scale on a 4-point scale (state: 1-not at all/4-very much so; trait: 1-almost never/4-almost always). Both scales were previously validated in Dutch samples: IRI^67^, and STAI (van der Ploeg, 2000). IRI was used here to measure perspective taking, empathic concern and other empathy related constructs that might influence perception of in- and outgroup individuals^5^. The STAI was used to measure the potential influence of state and/or trait anxiety on emotion perception.

### Design and Procedure

#### fMRI experiment

**Figure 3:**
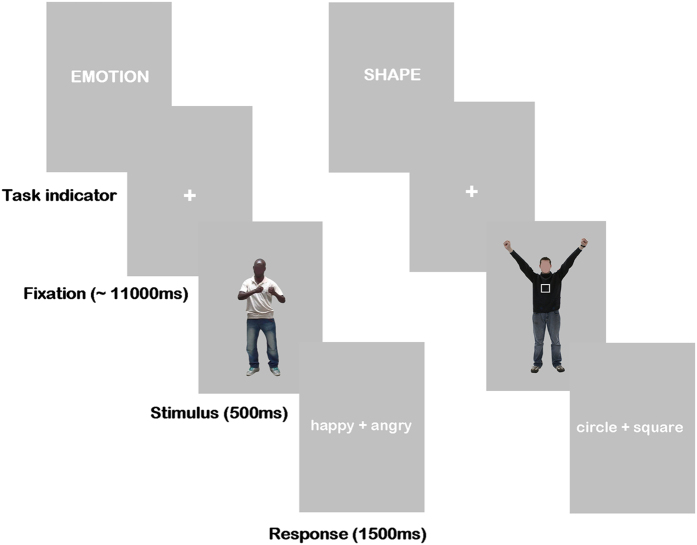
Examples of both emotion and shape task trials. Participants saw a screen indicating which task they were to perform, followed by a fixation period, one body, and a response window in which they indicated their answer via a button press on a response box. The index finger corresponded to the word on the left of the screen, and the middle finger the right, with response options being randomised to avoid motor preparation.

Participants were scanned using a Siemens 3 T Prisma scanner. Earplugs were used to attenuate scanner noise and padding was used to reduce head movements. All stimuli presented during the fMRI session were projected to a clear screen at the back of the scanner bore that participants could see using a mirror mounted on top of a head coil. Stimuli were presented sequentially in the centre of a grey screen. Participants completed two categorisation tasks that followed a mixed block/event related design of four separate runs. Body stimuli were viewed in a slow event related design across each run, with each run comprising of two pseudo-randomly ordered blocks: one emotion categorisation task, and one shape categorisation task, which were intended to act as explicit and implicit measures of emotion perception, respectively. In the ‘emotion’ block, participants indicated after each body presentation, via a response box, whether it was happy or angry; in the ‘shape’ block, they indicated whether a circle or square was superimposed on each body. The block type was indicated for 2 s before each block began. The trials in each block were separated by a fixation cross that appeared for 10 or 12 s (in a pseudo-random order). Following the fixation cross, a body appeared for 500 ms. This was then followed immediately by a response screen which appeared for 1500 ms, where the two response options appeared to the right and left of the fixation cross. The word to the left of the cross corresponded to the index finger on the response box, and the word to the right to the middle finger. Response options were randomised so to avoid motor preparation. Each body was presented twice in each run: once during emotion categorisation and once during shape categorization. Therefore, each run consisted of 40 trials (excluding task indicators). Illustrative examples of stimuli and the task design are presented in Fig. 3.

After the experiment, each participant was also administered with two questionnaires: the interpersonal reactivity index^65^ which provides a measure of empathy, and the State-Trait Anxiety Inventory which is commonly used as a measure of trait and state anxiety^66^.

#### Neuroimaging parameters, acquisition and preprocessing

A T2*-weighted gradient echo EPI sequence was used to acquire functional data covering the whole brain, with 2 × 2 × 2 mm3 resolution (64 slices without gaps, TR = 2000 ms, TE = 30 ms, flip angle = 77, multiband acceleration factor = 2, FOV = 160 × 160, matrix size = 100 × 100). Additionally, a T1-weighted MPRAGE sequence was used to acquire the anatomical structures (1 × 1 × 1 mm3, TR = 2300 ms, TE = 2.98 ms) for each participant. Pre-processing was done using BrainVoyager software (BrainVoyager, QX). Data from each run were motion-corrected by realigning to the first volume of the first run, and a two-cycle temporal high-pass filter was applied to remove low frequency linear and quadratic trends. Furthermore, a Gaussian spatial smoothing kernel of 6 mm was applied to data from each run.

### Analysis

#### fMRI experiment

Functional images were averaged across subjects according to the condition for each block in each run, and separate predictors assigned to each trial. For 7 participants, only three of the five original trials for each condition were included as predictors due to an initial error in stimulus presentation (i.e. Stimuli 1–3 within the white happy, white angry, black happy, and black angry conditions, for both the Emotion and Shape tasks). We then computed a group statistical map, calculated by using a random-effects model, restricting this by using a mask to exclude non-grey matter voxels. T-contrasts between Black and White bodies (both averaged across emotion, and separately for each emotion) were calculated firstly averaged across task, and then separately for the emotion categorisation and shape categorisation tasks.

The statistical threshold was set at p < 0.05, FDR cluster-level corrected. First, a single voxel threshold of p = 0.001 (uncorrected) was used for initial statistical maps. Next, a whole-brain correction criterion was calculated by estimating a false-positive rate for each cluster. This was established by means of Monte-Carlo simulation (1000 iterations), with the minimum cluster size threshold applied to the statistical maps corresponding to a cluster-level false-positive rate (α) of 5%. Cluster size is reported in number of anatomical voxels.

Additionally, we also conducted a targeted region-of-interest analysis within the bilateral amygdala in order to investigate whether there was an overall difference in this region between black and white bodies. The amygdala mask was taken from the Statistical Parametric Mapping library and transposed to the BrainVoyager interface. Here, results were also set at p < 0.05 FDR cluster-level corrected, but with an input voxel threshold of p = 0.01 (uncorrected). Finally, we also performed a correlation analysis between activity in the amygdala and both IAT and STAI scores, in order to investigate whether either empathy or anxiety was related to race-related activity in this region.

#### On-line behavioural data

Participant accuracy was analysed for both the shape and emotion tasks. Only the first three trials per condition within both the shape and emotion tasks were included for 7 participants (see above). For the emotion task, we calculated the accuracy for each condition (i.e. Black Angry, Black Happy, White Angry, White Happy), with data being averaged across the four runs. For the shape task, we calculated only the overall accuracy, averaged across all conditions and the four runs, due to the task being unrelated to emotion categorisation. Here, the main interest was to ensure that in the implicit task participants were still focussed on the stimuli.

## Additional Information

**How to cite this article**: Watson, R. and Gelder, B. How white and black bodies are perceived depends on what emotion is expressed. *Sci. Rep.* **7**, 41349; doi: 10.1038/srep41349 (2017).

**Publisher's note:** Springer Nature remains neutral with regard to jurisdictional claims in published maps and institutional affiliations.

## Figures and Tables

**Table 1:** Whole brain race effects (i.e., black bodies vs. white bodies; white bodies vs. black bodies), averaged across both tasks.

Brain Region	L/R	x	y	z	t value	p value	Cluster size
EMOTION + SHAPE CATEGORISATION TASK							
Black bodies vs. White bodies							
Inferior Parietal Lobule	L	−59	−27	28	4.707546	0.000135	95
White bodies vs. Black bodies							
No significant voxels							
Black angry bodies vs. White angry bodies							
No significant voxels							
White angry bodies vs. Black angry bodies							
Fusiform Gyrus	R	43	−45	−12	4.60814	0.000170	137
Black happy bodies vs. White happy bodies							
Postcentral/precentral gyrus	R	49	−15	30	6.0447	0.000007	367
	R	35	1	30	5.573438	0.000019	1082
	R	49	1	10	5.412478	0.000027	356
	L	−33	7	32	4.736381	0.000126	179
	L	−25	−37	62	6.079047	0.000006	400
	L	−53	−33	50	7.003599	0.000001	1806
	L	−49	−31	36	5.824973	0.000011	825
Superior parietal lobule	R	17	−67	54	5.177658	0.000046	110
Inferior semi-lunar lobule	L	−21	−67	−38	5.374045	0.000029	242
Precuneus	L	−33	−67	40	4.334905	0.000322	92
Superior/middle frontal gyrus	R	5	9	50	7.406028	0.000001	1497
	R	27	−7	60	6.956295	0.000001	650
Inferior/Middle occipital gyrus	L	−43	−73	−8	9.440401	0.000001	4401
	L	−31	−77	16	4.851936	0.000097	90
	R	33	−79	22	4.803578	0.000108	202
	L	−37	−81	8	4.907144	0.000085	103
Superior occipital gyrus	L	−33	−83	30	5.538504	0.000020	163
Lingual gyrus	L	−15	−75	2	4.949526	0.000077	178
Angular gyrus	L	−41	−63	36	5.153766	0.000048	144
Fusiform gyrus	L	−33	−39	−16	5.823625	0.000011	91
Cerebellum	L	−41	−51	−24	4.585514	0.000179	228
Cerebellum/Fusiform gyrus	L	−43	−45	−18	4.41007	0.000270	90
Claustrum	R	29	23	8	5.268699	0.000037	264
Insula	L	−35	17	4	4.737395	0.000126	328
	L	−5	−27	−38	6.319092	0.000004	140
White happy bodies vs. Black happy bodies							
No significant voxels							

The peak voxel of each significant cluster is reported; cluster size is reported in anatomical voxels. Results are thresholded at p < 0.05 FDR corrected for cluster size.

**Table 2:** Whole brain race effects within the emotion categorisation task.

Brain Region	L/R	x	y	z	t value	p value	Cluster size
EMOTION CATEGORISATION TASK							
Black bodies vs. White bodies							
Insula	R	33	23	10	6.167003	0.000005	349
Superior frontal gyrus	L	−1	5	56	5.341044	0.000032	192
Medial frontal Gyrus	L	−3	17	44	4.149449	0.000496	439
White bodies vs. Black bodies							
Parahippocampal Gyrus*	R	25	−11	−16	5.884	0.000009	192
Black angry bodies vs. White angry bodies							
No significant voxels							
White angry bodies vs. Black angry bodies							
No significant voxels							
Black happy bodies vs. White happy bodies							
Inferior frontal gyrus	R	45	9	28	5.822988	0.000011	153
	L	−37	7	28	5.22151	0.000041	317
Insula	R	31	23	8	6.691979	0.000002	983
	L	−37	19	6	4.964108	0.000075	749
Superior frontal gyrus	R	3	7	50	6.023604	0.000007	1004
Middle occipital gyrus	L	−47	−59	−8	5.70624	0.000014	126
White happy bodies vs. Black happy bodies							
No significant voxels							

The peak voxel of each significant cluster is reported; cluster size is reported in anatomical voxels. Results are thresholded at p < 0.05 FDR corrected for cluster size. * Cluster extended to hippocampus and amygdala.

**Table 3:** Whole brain race effects within the shape categorisation task.

Brain Region	L/R	x	y	z	t value	p value	Cluster size
SHAPE CATEGORISATION TASK							
Black bodies vs. White bodies							
Thalamus	R	13	−31	12	5.49698	0.000022	77
White bodies vs. Black bodies							
No significant voxels							
Black angry bodies vs. White angry bodies							
No significant voxels							
White angry bodies vs. Black angry bodies							
Precentral gyrus	R	43	−1	34	6.899713	0.000001	570
Posterior cingulate	L	−23	−63	10	5.129292	0.000051	125
Precuneus	L	−23	−79	38	5.12979	0.000051	123
Medial frontal gyrus	L	−3	7	44	5.215328	0.000042	345
Middle frontal gyrus	L	−17	33	44	4.510719	0.000213	83
Middle occipital gyrus	L	−31	−83	16	5.562295	0.000019	497
	L	−51	−61	−4	6.81547	0.000001	990
Fusiform gyrus	R	41	−45	−12	5.741293	0.000013	796
Superior temporal gyrus	L	−51	−49	12	5.267976	0.000037	91
Black happy bodies vs. White happy bodies							
Superior/middle temporal gyrus	R	51	−21	0	5.497991	0.000022	1091
	R	45	−63	0	4.675128	0.000146	102
	L	−49	−45	12	4.662863	0.000150	166
	L	−57	−39	6	4.568361	0.000187	101
Supramarginal gyrus	R	57	−47	22	5.118039	0.000052	329
Precuneus	R	9	−73	40	4.701671	0.000137	279
	R	1	−65	38	4.744387	0.000124	132
	L	−9	−69	40	4.753948	0.000121	131
	L	−15	−69	24	5.357591	0.000030	137
	L	−23	−71	30	4.705309	0.000136	116
Cuneus	R	21	−81	16	4.748688	0.000123	550
Superior parietal lobule	R	15	−65	54	5.111515	0.000053	192
	R	27	−61	42	4.950864	0.000077	138
Inferior parietal lobule	L	−43	−29	42	4.715022	0.000133	156
	L	−37	−43	44	5.826255	0.000011	227
	L	−43	−45	50	5.566172	0.000019	202
Paracentral lobule	L	−3	−19	46	5.2994	0.000035	160
	L	−5	−37	50	5.236001	0.000040	210
Postcentral gyrus	L	−25	−39	60	4.68247	0.000143	214
Middle occipital gyrus	L	−41	−67	−6	8.727465	0.000001	4363
	R	33	−79	2	5.523646	0.000021	1067
	L	−31	−83	0	4.638104	0.000159	229
	L	−35	−81	8	5.040009	0.000063	91
Lingual gyrus	R	33	−73	−8	8.154386	0.000001	981
Superior occipital gyrus	L	−31	−83	32	5.056104	0.000060	229
Cerebellum	R	17	−47	−8	4.83826	0.000100	146
	L	−19	−47	−10	4.890746	0.000088	186
Cingulate gyrus	L	−3	−1	42	4.995483	0.000069	595
	L	−3	−33	30	4.792445	0.000111	183
White happy bodies vs. Black happy bodies							
No significant voxels							

The peak voxel of each significant cluster is reported; cluster size is reported in anatomical voxels. Results are thresholded at p < 0.05 FDR corrected for cluster size.

**Table 4:** Amygdala specific race effects.

Brain Region	L/R	x	y	z	t value	p value	Cluster size
EMOTION + SHAPE CATEGORISATION TASK							
White bodies vs. Black bodies							
Amygdala	R	25	−9	−18	5.433138	0.000026	126
EMOTION CATEGORISATION TASK							
Amygdala	R	25	−11	−16	5.883995	0.000009	129
	R	15	−7	−14	4.002468	0.000699	68

The peak voxel of each significant cluster is reported; cluster size is reported in anatomical voxels. Results are thresholded at p < 0.05 FDR corrected for cluster size.
